# The effect of music familiarity on preschoolers’ heart rate during musical engagement: preliminary report

**DOI:** 10.3389/fpsyg.2026.1699832

**Published:** 2026-02-11

**Authors:** Diana R. Dansereau, Kaitlyn Leahy, Jack Carbaugh

**Affiliations:** Boston University College of Fine Arts School of Music, Boston, MA, United States

**Keywords:** attention, heart rate, heart rate variability, musical engagement, novelty, physiological response, preschoolers, young children

## Abstract

Young children’s heart rates (HR) have been shown to decrease, and heart rate variability (HRV) has been shown to become suppressed, in response to new stimuli. This response has been documented when children experience novel physical objects as well as novel aural stimuli, giving cause to wonder whether novel musical stimuli would be accompanied by cardiovascular changes. The purpose of this study was to (1) document children’s HR and HRV in a naturalistic setting (early childhood music classes), (2) conduct analyses to ascertain whether HR and HRV are associated with the level of familiarity of musical material among study participants, and (3) draw recommendations for follow-up research based on methods used in this preliminary study. Seven children, ages 3–5, engaged in 30-min music classes, once-per-week for eight consecutive weeks, while wearing heart rate monitors. Findings included a notable synchronization of heart rate across children and in response to the musical content of classes. Analysis of variance revealed that heart rates during unfamiliar music were significantly lower than during transitions or familiar music; however, a random effects linear panel model did not detect a significant difference between the heart rate means in the varying familiarity/transitional states. Recommendations for follow-up research include employing a more complex analytical model in order to better capture the serial correlation and moving trend of the heart rate data, incorporating technology that produces continuous heart rate tracking and can be employed in naturalistic settings, and engaging with a larger sample in order to determine the extent to which changes in heart rate during music classes may be associated with the familiarity of the musical material.

## Introduction

Engagement with music has been shown to correlate with various extramusical cognitive functions. These functions include language skills such as sensitivity to speech rhythm (Magne et al., 2016), and executive functions such as inhibitory control (Bialystok and Depape, 2009) and working memory (Bergman Nutley et al., 2014; Slevc et al., 2016). Given this, it can be assumed that important neural and physiological processes are at play during musical engagement, and that there may be shared neural processes between extramusical and musical cognitive functions (Gordon and Magne, 2017). One such musical cognitive function is *audiation*, in which an individual mentally holds, replays, and understands sounds (Gordon, 2013; Runfola and Taggart, 2005). Through audiation, “sound becomes music and meaning is attributed to that music” (Grinspun et al., 2020, p. 2). The ability to audiate is theorized to be concomitant with music aptitude, and therefore directly and positively related to one’s achievement in music (Gordon, 2013).

A key component of audiation is attention. Specifically, selected attention and inhibitory control have been shown to correlate with audiation (Grinspun et al., 2020), and inhibiting attention to irrelevant information is essential for audiation (Gordon, 2007). According to Grinspun et al. (2020), “for audiation [to occur] it is necessary to maintain the focus of attention and remain alert during relatively long periods of time” (p. 6). Unlike the cognitive process of audiation, attention and alertness can be observed, and indeed, focused or fixed attention has been documented while young children were engaged in musical tasks within a learning context (Dansereau and Wyman, 2020). Specifically, children were observed “ceasing all movement, staring into the distance without a specific [visual] focus, showing intense facial affect, and sometimes displaying an open mouth and/or tilted head” (Dansereau and Wyman, 2020, p. 26). This description is similar to descriptions of *audiation stare*—which is sometimes characterized by an open mouth and tilting of the head. According to Gordon (2013), audiation stare is present at “the first glimpse of discrimination, the realization sounds of music can be same or different” (p. 111).

Noting the potential shared neural processes between musical and extramusical cognitive abilities (Gordon and Magne, 2017), such fixed attention may be akin to the focused attention infants have demonstrated when shown novel physical objects or visual stimuli––a response that has been accompanied by a decrease in heart rate (Lansink and Richards, 1997) and a suppression of heart rate variance (Richards and Casey, 1991). Though musical engagement involves ‘sonic’ objects rather than physical or visual objects, it may be that cardiovascular changes also accompany focused attention on novel music, potentially indicating audiation. Such indicators would not only provide psychophysiological evidence that young children are indeed attending to the sonic object, but they could offer information regarding the specific musical elements that are being perceived as novel and thus triggering cardiovascular changes. These data would shed additional light on young children’s music perception and cognition tendencies, and could ultimately allow for the evaluation of the effectuality of musical engagement in encouraging audiation. Consequently, in this preliminary study, we sought to determine whether attention to unfamiliar musical stimuli is accompanied by changes in heart rate (HR) and/or heart rate variance (HRV) among a group of preschoolers––a phenomenon that has yet to be studied. Specifically, the purpose was to (1) document children’s HR and HRV in a naturalistic setting (early childhood music classes), (2) conduct analyses to ascertain whether HR and HRV are associated with the level of familiarity of musical material among study participants, and (3) draw recommendations for follow-up research based on the methods used in this preliminary study.

## Literature

One of the first studies to document that young children exhibit a physiological response when encountering novel physical objects was by Hughes and Hutt (1979), who tracked the physiological responses of 2-year-olds engaging with an unfamiliar toy. The researchers found that the children’s heart rate variability (HRV)—defined as the fluctuation in time between adjacent heart beats (Shaffer and Ginsberg, 2017)––was suppressed while the children engaged with the object, and that the degree of suppression was proportional to the mental load and level of attention involved (Hughes and Hutt, 1979). Further, they discussed differences between exploration and play with a novel object, noting that “exploration occurs in the presence of stimulus novelty, incongruity, etc., whereas playful activity occurs in conditions of familiarity and states of relative relaxation” (Hughes and Hutt, 1979, p. 254). They reported that children’s HRV was more suppressed during periods of exploration than during periods of play with an object.

Similar to HRV, Hughes and Hutt (1979) also noticed a decline in heart rate (HR)—defined as number of beats per minute (Shaffer and Ginsberg, 2017)––associated with exploration of a novel object, though this decline was not statistically significant in their study. Since then, however, researchers have consistently documented such decreases in heart rate and linked them with an evolutionary orienting response to a stimulus. This cardiovascular response likely allows for improved intake of the novel stimulus (Andreassi, 2010), and it has been documented across varying contexts and with differing technologies (Chuen et al., 2016; Mojtabavi et al., 2020; Proverbio et al., 2015; Stekelenburg and Van Boxtel, 2002). For example, infants demonstrated a significant deceleration of HR when exposed to evolutionary fear-relevant sounds such as thunder or angry voices (Erlich et al., 2013). Although humans process noise differently than music (Gomez and Danuser, 2004), musical stimuli have also been found to initiate this cardiovascular orienting response in both infants and adults (Chuen et al., 2016; Garunkstiene et al., 2014; Proverbio et al., 2015; Witvliet and Vrana, 2007). For example, adults’ HRV was determined to be lower upon introduction of a musical stimulus than when the musical material became familiar (Kirk et al., 2022). Similarly, HR has been shown to be influenced by the familiarity of music, in that adults have demonstrated quicker HR deceleration with novel music than with music to which they have become accustomed (Witvliet and Vrana, 2007).

Scholars have also investigated cardiovascular responses during periods of attention. For example, researchers have found that attention in infants corresponded with decreased HR in response to novel physical objects (Lansink and Richards, 1997) and suppressed HRV in response to a visual stimulus (Richards and Casey, 1991). Further, when accompanied by attention, HR has been found to decelerate for approximately 5 s when infants were presented with visual stimuli (Richards and Casey, 1991) or foreign lullabies (Bainbridge et al., 2021).

Though cardiovascular changes when encountering musical stimuli have been documented among infants and adults, possible effects have yet to be studied in young children. Further, previous studies have been conducted exclusively in laboratory settings. Research on young children’s cardiovascular responses to music within a naturalistic setting would be illuminative for those concerned with children’s attention to and perceptions of music during music classes. It may also offer insight on the psychophysiological underpinnings of the fixed attention that has been documented while young children are engaged in musical tasks within a naturalistic learning context (Dansereau and Wyman, 2020).

## Materials and methods

This study took place at a childcare center on a university campus in a large city in the northeastern region of the United States. Seven children, ranging in age from 44 months to 69 months at the onset of the study participated. The children, two girls and five boys, comprised a single multi-age class (for children ages 3–5 years) within the center. The group of participants was racially heterogeneous and none of the children had been identified as requiring special learning services. All study procedures were conducted under the auspices of the university’s Institutional Review Board. The children’s parents provided written consent and the children provided verbal assent to participate in the study.

The participants engaged with the first author in 30-min music classes, once-per-week for 8 weeks. We drew upon studies on the effects of musical engagement on young children (Dansereau, 2011; Ritblatt et al., 2013) to determine the content and pedagogical approach of the classes. During classes, the first author modeled musical behaviors and offered opportunities for the children to respond, engaging in as many one-on-one musical serve-and-return (Center on the Developing Child at Harvard University, 2007) interactions as possible. Songs and chants were repeated multiple times successively within a class and also across the weekly classes. Silences were purposefully included both within songs and chants, as well as between repetitions to offer children time to audiate and anticipate upcoming aural stimuli (Valerio et al., 1998). The musical material was selected to provide heterogeneity in terms of musical characteristics. As such, it represented multiple modes, meters, and tempi, and was texted and untexted (Table 1). The first author minimized the use of speaking voice during class time to prioritize musical sound and avoid interrupting audiation. Further, we analyzed musical selections to ensure gender equity and avoid heteronormative themes. The first author also incorporated beat-centered and flow movements throughout.

**Table 1:** Characteristics of musical material.

Title of musical material	Type	Mode	Meter	Text	Tempo (approximate)
A Shake	Song	Major	Duple	Texted	64 bpm
Alley Balley	Song	Major	Duple	Texted	71 bpm
Goldfish	Song	Dorian	Triple	Untexted	59 bpm
Hello song	Song	Mixolydian	Duple	Texted	58 bpm
Hello, hello, hands	Song	Mixolydian	Duple	Texted	65 bpm
Hey, Goodbye	Song	Mixolydian	Duple	Texted	68 bpm
I Am a Narwhal	Song	Major	Duple	Texted	64 bpm
Jeremiah Blow the Fire	Song	Major	Duple	Texted	62 bpm
Lavender’s Blue	Song	Major	Triple	Untexted	40 bpm
Little Jenny Brown	Song	Minor	Duple	Texted	63 bpm
Mr. Turkey, Mrs. Duck	Chant	N/A	Duple	Texted	70 bpm
Open, Shut Them	Chant	N/A	Duple	Texted	67 bpm
Peek-a-boo	Sol-Do Tonal Patterns	N/A	N/A	Texted	N/A
Red Umbrella	Song	Dorian	Unusual	Untexted	92 bpm
Smooth Road	Chant	N/A	Duple	Texted	82 bpm
Snowflake	Chant	N/A	Triple	Untexted	80 bpm
Stretch and Bounce	Chant	N/A	Duple	Untexted	84 bpm
Two Little Birds	Song	Major	Duple	Texted	56 bpm
Walk and Stop	Song	Major	Duple	Texted	56 bpm
When You’re One	Song	Major	Duple	Texted	100 bpm
Where is [name]?	Song	Major	Duple	Texted	62 bpm
Wiggle Song	Song	Dorian	Duple	Texted	69 bpm

## Data collection

During the data collection process while the first author led classes, the second author set up, maintained, and monitored equipment. The children wore Polar H10 HR monitors (Polar Electro, 2019) which are attached to non-slip, adjustable elastic belts that are positioned around the chest. Polar’s wearable devices have been used previously in research settings involving young children (e.g., de Souza et al., 2019; McGowan et al., 2021) and have been shown to produce similar results to an electrocardiogram (ECG) (Dobbs et al., 2019). The heart rate data collected were transmitted to an iPad in real-time and were later inputted into a spreadsheet in three-second intervals, resulting in 28,995 data points. In addition to heart rate, the events of the music classes were recorded with a 360fly, 360-degree camera (360fly, Inc., 2019). We reviewed and analyzed video recordings, and coded each activity to indicate whether it consisted of musical material that was familiar or unfamiliar to the children. In addition, we identified periods of time when no musical stimuli were presented and labeled these periods as transitions. This coding allowed us to analyze for changes in HR and HRV according to the familiarity of musical stimuli.

## Analysis

We began analysis with a lengthy period of visualizing the time series data in order to obtain an initial understanding of how the heart rates changed across different episodes and instances of familiar or unfamiliar musical content. We visualized the data in raw form, pooled, and with the use of box plots. We then conducted a change-point analysis, which allowed us to locate the specific times in which statistically significant changes in mean heart rate occurred. Change-point analysis is fairly robust, with the only assumption being an independent error structure (Shi et al., 2022). Due to the time series being non-stationary, this assumption was not met. Consequently, we employed nonparametric methods for change-point detection of the mean heart rate. For each week’s data, the children’s individual data were mean-centered and pooled together. We used the R package changepoint.np––the nonparametric add-on to changepoint––and ran change-point analysis using CROPS penalty (Haynes et al., 2014) and the PELT algorithm (Killick et al., 2012).

Heart rate variability is typically measured using medical evaluation technology (e.g., electrocardiograms) that produces continuous heart rate tracking and allows for the analysis of the time lapses between heart beats. Due to the naturalistic setting of this study as well as funding limitations, the use of such technology was not feasible. Consequently, we calculated the variance of heart rates within activities or transitions as an estimate of heart rate variability.

For change-point detection of the variance, nonparametric methods were not widely available; however, due to the robust nature of change-point analysis, a minor break in assumption for analyzing variance has been shown to be mostly inconsequential. The change-point analysis of the variance proceeded largely the same as the change-point analysis of the mean. The main distinction was that we analyzed the first difference of the heart rates, as this removed the trend in the data that can obfuscate changes in variance. The data were then assumed to be independent with a normal distribution. Once a change-point was detected, we checked if it aligned with a change in episode or familiarity.

Our next step was to perform a one-way analysis of variance (ANOVA) to check for differences in the mean HRs during episodes that were familiar, unfamiliar, or transitional. The first assumption underlying ANOVA is independence among the sample draws (Navarro, 2022). Because this independence does not exist within time correlated data such as heart rates, we pooled the data by episode, which collapsed much of the time correlation. We contend that it is logical to conclude that mean heart rates were independent between episodes. The other two assumptions of equal variance between groups, and the normal distribution of data were met.

Finally, to further explore possible effects of familiar musical stimuli on HR, we performed a linear panel model. Because the data were within a panel or longitudinal format, as heart rates were observed for multiple subjects over the same periods of time, a time variable needed to be built for the panel data. This variable gave each observation per subject a chronological value. A null value was assigned if a child was not present during a given week, or if heart rate could not be monitored. Indicator variables were used for the familiarity conditions, with the unfamiliar condition as the baseline.

## Results

Average heart rate during data collection varied across participants and ranged from 103.43 to 121.70 with a grand mean of 112.16 (Table 2). Within each class, and pooling the children’s heart rates together, the average heart rates ranged from 108.32 (in Week 5) to 122.53 (in Week 4) with a grand mean of 112.78 (Table 2). We also calculated mean heart rates recorded during each of the 21 songs/chants that the children experienced over the 8 classes. The lowest mean heart rate was observed during the song “I am a Narwhal” (Arrow, 2019) and the highest was observed during the song “A Shake” (Bolton, 2010).

**Table 2:** Mean heart rates, standard deviations, and variances by child and week.

Child/Week	Child/Week number	*M*	*SD*	Var
Child	1	114.24	10.13	102.52
	2	116.69	9.98	97.53
	3	108.18	9.20	84.63
	4	121.70	14.36	206.31
	5	113.84	8.85	78.34
	6	103.43	10.99	120.68
	7	107.06	10.28	105.66
Grand mean	112.16		99.46	
Week	1	110.90	9.95	98.93
	2	110.79	9.41	88.62
	3	115.08	10.22	104.52
	4	122.53	13.22	174.81
	5	108.32	9.93	98.51
	6	115.51	14.42	208.02
	7	109.12	11.81	139.46
	8	110.01	10.98	120.50
Grand mean	112.78		129.17	

In terms of heart rate variability, participants’ heart rate variances ranged from 78.34 to 206.31 with a grand mean of 99.46 (Table 2). Within each class, and pooling children’s heart rate variances together, variances ranged from 88.62 (in Week 2) to 208.02 (in Week 6) with a grand mean of 129.17 (Table 2). As with heart rate, we calculated mean variances during each of the songs/chants that the children experienced over the 8 classes. The lowest variance occurred during the song “Goldfish” (Valerio et al., 1998) and the highest variance occured during “Hello” (Bolton, 1999).

### Visualizations

**Figure 1:**
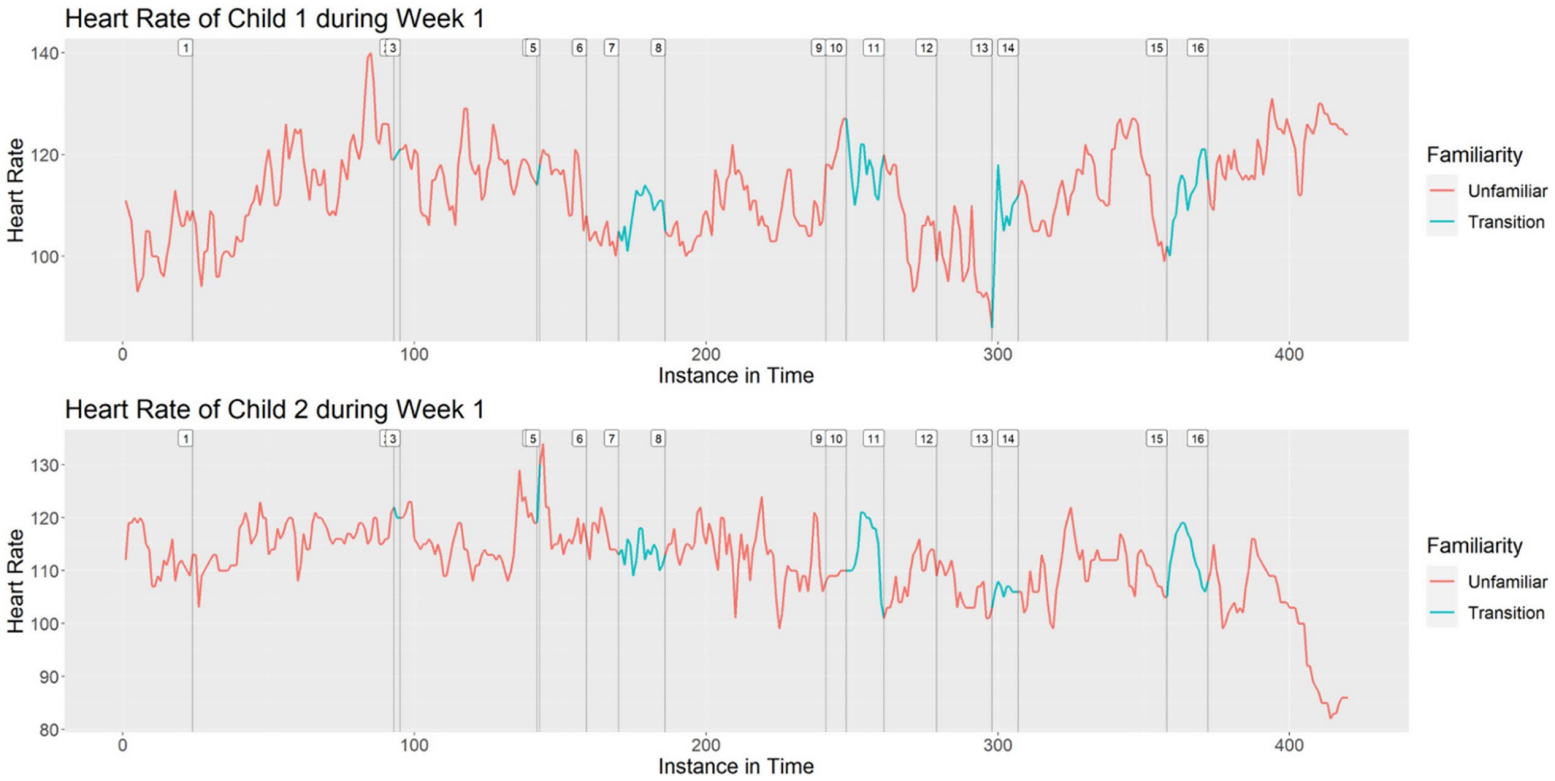
Heart rates of Child 1 and Child 2 during Week 1.

**Figure 2:**
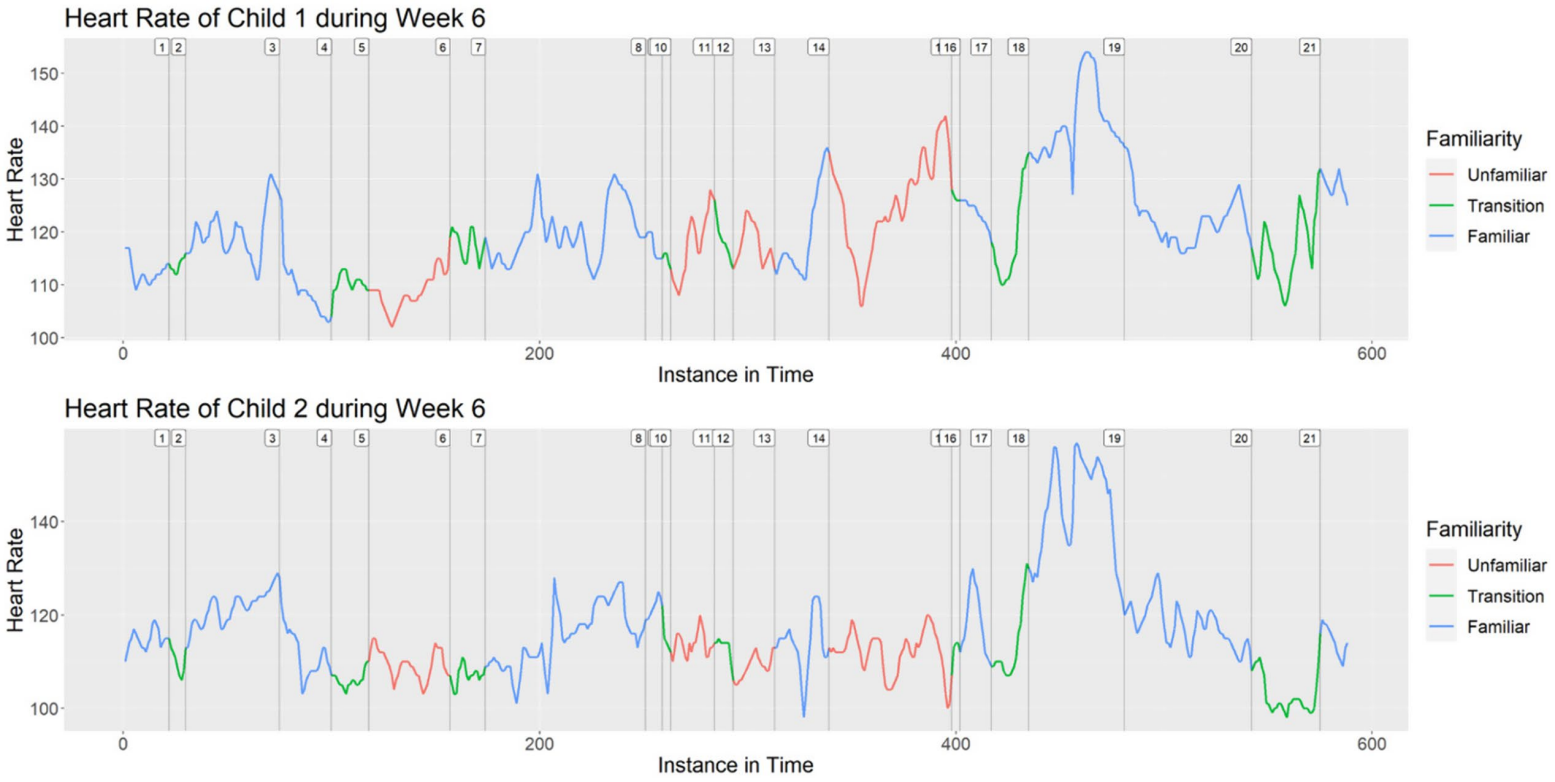
Heart rates of Child 1 and Child 2 during Week 6.

As an example of the visualizations that we created during the initial phase of analysis, Figure 1 shows the heart rates of Child 1 and Child 2 during Week 1.^1^^,^^2^ Figure 2 shows the heart rates of the same two children during Week 6, with indications that the majority of the material was familiar to the children at this stage of data collection.

**Figure 3:**
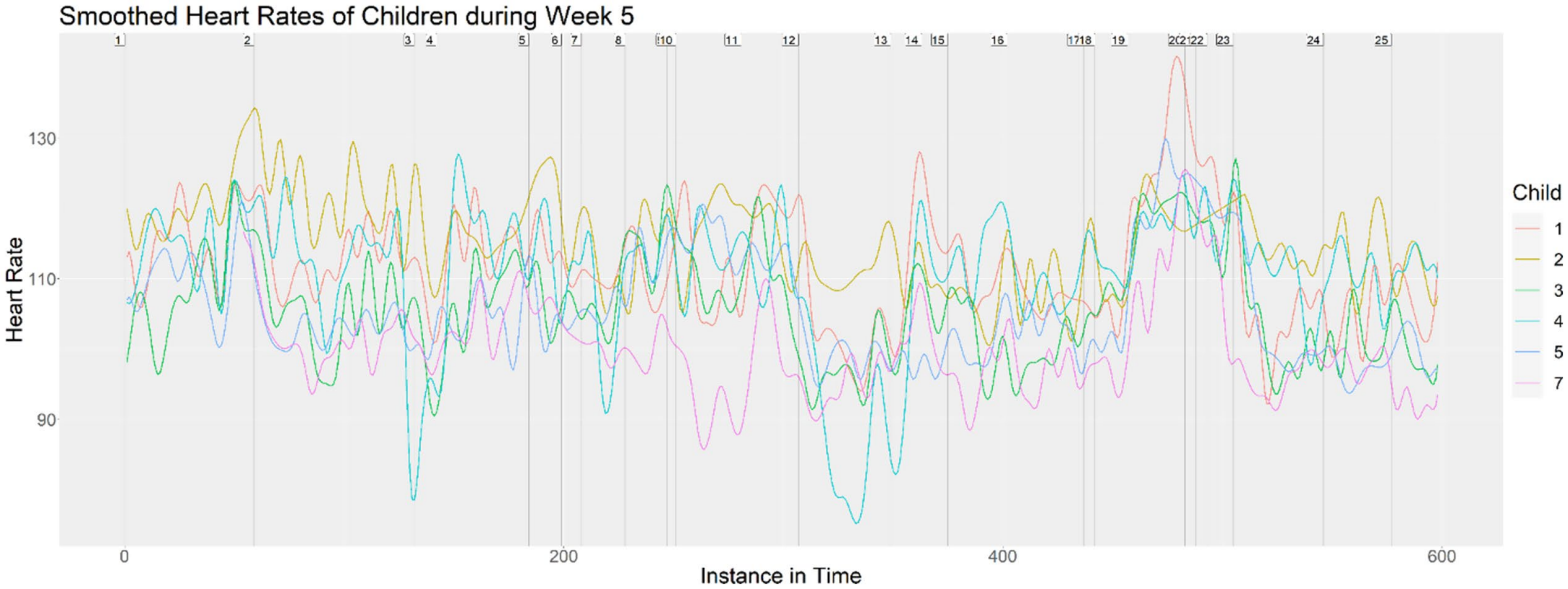
Heart rates of children during Week 5.

**Figure 4:**
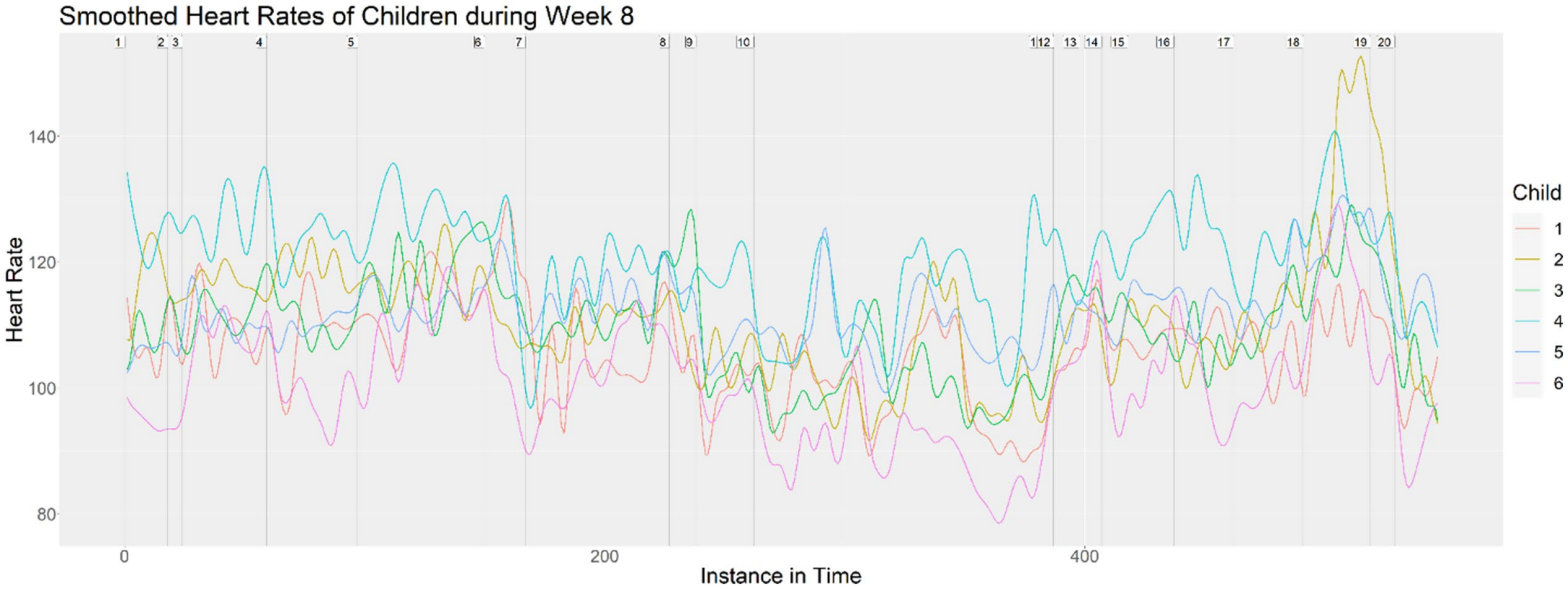
Heart rates of children during Week 8.

**Figure 5:**
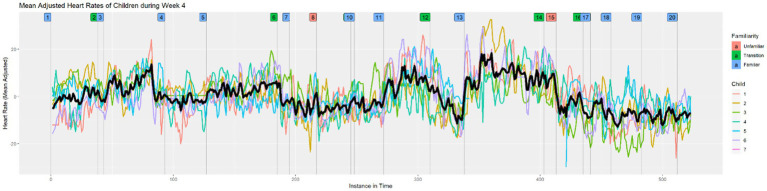
Mean adjusted heart rates of children during Week 4.

Next, we layered all seven children’s heart rates onto the same visualization for each class to see the relationship among the heart rates that may have indicated a response to the musical material. As examples, Figures 3, 4 show the layered heart rates for children during Week 5 and Week 8. Figure 5 shows the mean-adjusted heart rates for all children in Week 4, with a black curve indicating the pooled average.

### Change points

Change point analysis revealed 85 significant changes in mean heart rate across the 8 weekly music classes. The lowest number of change points (8) occurred in Week 5, and the most change points (12) occurred in Weeks 1, 3, and 7. The change points of HRV (variance) were less common than those for the mean, and highlighted segments that were more localized. This was a general pattern across all weeks; it appeared that the variance remained constant throughout the weekly classes, with only small areas of abnormally high or low variance. These change-points also aligned less frequently with the changes in episode. Given the constancy of the HRV across weeks, we elected to proceed with analysis of HR only.

**Figure 6:**
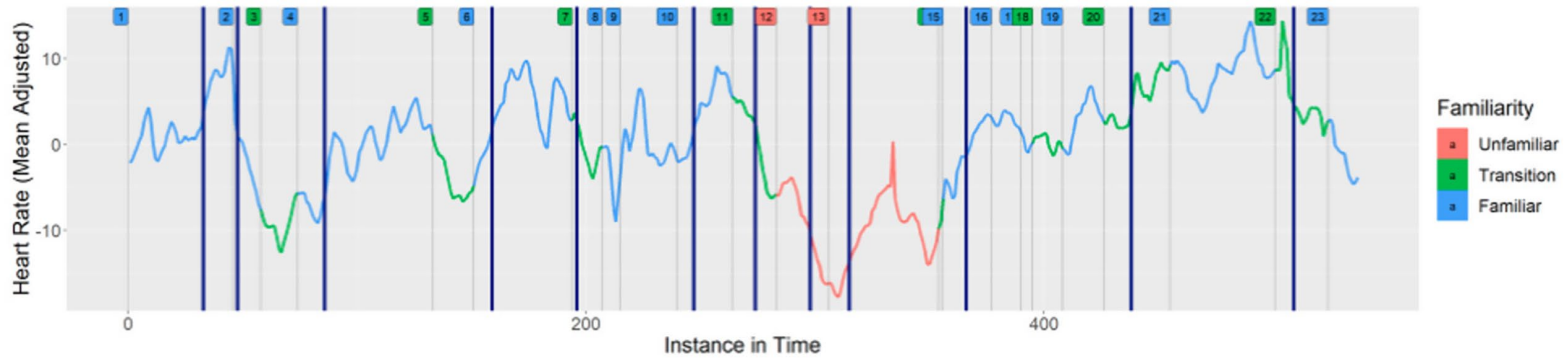
Change points of the mean for the average heart rate trend during Week 7.

We plotted the change points as well as the particular musical activities on the graph of heart rates for each week, and then classified the musical activities according to familiarity. As an example, Figure 6 depicts the mean heart rate during Week 7, color coded according to familiarity, with the significant change points marked by vertical navy-blue lines.

**Figure 7:**
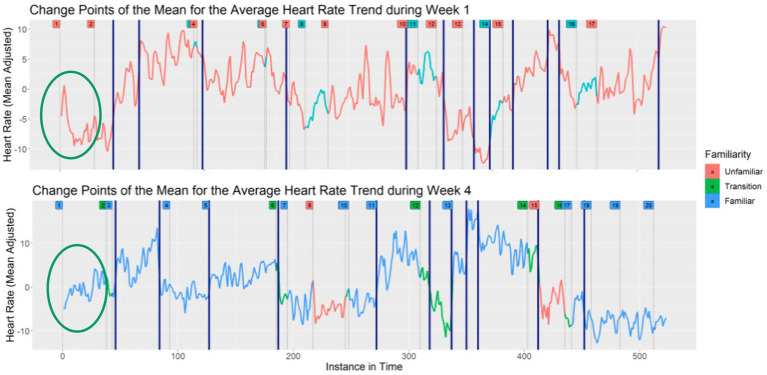
Change points of the mean for the average heart rate trend during Weeks 1 and 4.

This process allowed us to observe changes or similarities in heart rate across weeks. For example, in Figure 7 the green circles indicate when the first author led the singing of “Hello” in Week 1 and in Week 4. In Week 1, we classified the song as unfamiliar, but then classified it as familiar in Week 4. A drop in the children’s heart rate occurred during the initial presentation of that song in Week 1. In Week 4, when the song was quite familiar, there was no initial drop, but rather an increase in heart rate.

### Analysis of variance

Analysis of variance on the mean heart rate for unfamiliar musical content (*M* = 109.44), familiar musical content (*M* = 113.98), and transitions (*M* = 112.08) showed statistically significant differences [*F*(2,159) = 4.879, *p* = 0.009]. Tukey *post hoc* testing revealed that the heart rates during unfamiliar music were significantly lower than both the transition (*p* = 0.024) and familiar (*p* = 0.007) means. There was not a significant difference between the transition and familiar groups.

### Random effects linear panel model

Estimates, standard errors, and *p*-values are shown in Table 3 and random effect coefficient estimates appear in Table 4. These coefficient estimates depict the average increase in heart rate between a given familiarity condition and the baseline unfamiliar state, assuming that all else is held constant.

**Table 3:** Coefficient estimates.

Coefficient	Estimate	Standard error	*t*-stat	df	*p*-value
Transition	0.43	0.38	1.12	5.92	0.308
Familiar	0.90	0.50	1.78	5.91	0.126

**Table 4:** Random effects.

Child	Random effect
1	2.02
2	4.37
3	−3.91
4	9.20
5	1.68
6	−8.49
7	−4.87

According to the random effects linear panel model, heart rates during familiar music were on average not significantly higher than those during unfamiliar music, nor were heart rates during transitions significantly different during unfamiliar music.

After no significant result was found using the linear panel model, we determined the unexpectedness of the outcome by calculating the *S*-value (Mansournia et al., 2022). For a *p* value of 0.126, which was found for the difference between the means of the familiar and unfamiliar episode heart rates, the *S*-value is 2.98. This indicates that there are 2.98 bits of evidence against the null hypothesis, which suggests that the lack of significant difference between heart rates during familiar and unfamiliar music was about as unexpected as obtaining the same result across three fair coin tosses. Such a result is fairly uncommon, but not completely unlikely; hence, a similar interpretation may be adopted in regards to the model results.

## Conclusions and discussion

### Study purpose 1: document children’s HR and HRV in a naturalistic setting

Study procedures resulted in 28,995 data points documenting the children’s HR while engaged in early childhood music classes. Visualization of these data revealed a noticeable alignment of heart rates across children and in response to the musical content of the classes. Despite the children having different mean heart rates, the heart rates appeared to increase and decrease in tandem.

The highest HR was observed during the song “A Shake” (Bolton, 2010). This was unsurprising as there were two playful elements layered onto the singing of the song: shaker eggs and locomotor movement. Because this was the only activity that involved locomotor movement, it may be logical to conclude that the higher recorded HR of the children was due to the difference in physical activity (Rowlands et al., 1997). Conversely, the lowest mean heart rate was observed during the song “I am a Narwhal” (Arrow, 2019). The first introduction to this song was in the penultimate week of classes. In the final week, the first author played the song with a guitar, adding an additional layer of novelty. Therefore, it is possible that the low recorded HR during “I am a Narwhal” (Arrow, 2019) may have been in response to the children encountering this song as a largely novel sonic object, in combination with encountering a somewhat novel physical object (the guitar) (Hughes and Hutt, 1979).

### Study purpose 2: ascertain whether HR and HRV are associated with the level of familiarity of musical material among study participants

Analysis of variance with Tukey *post hoc* testing revealed that heart rates during unfamiliar music were significantly lower than both the transition and familiar means; however, the coefficient estimates and their standard error adjustments from the random effects linear panel model did not detect a significant difference between any of the heart rate means in the varying familiarity/transitional states. *S*-value analysis indicated that this result of no significant difference between heart rates during familiar and unfamiliar music was somewhat unexpected. In summary, the results of this study were not entirely conclusive regarding whether the children’s heart rates responded consistently and in reaction to the familiarity of the musical stimuli. As a result, it is not yet clear whether the young children’s responses to unfamiliar music were consistent with research showing a drop in heart rate when encountering unfamiliar physical objects (Hughes and Hutt, 1979; Lansink and Richards, 1997). More research is needed to determine the extent to which changes in heart rate during music classes are associated with the level of familiarity of the musical material and the attention necessary for audiation to occur.

### Study purpose 3: draw recommendations for follow-up research based on methods used in this preliminary study

Future research may involve a more complex analytical model in order to better capture the serial correlation and moving trend of the heart rate data. State-space models or neural networks, which can provide much greater flexibility in modeling the data, could be used as robust alternatives for checking differences in heart rates across a variety of factors. One limitation of this study is that the analysis of the familiarity groups still had the potential for time correlation and resulting errors. The use of state-space models may address this limitation as well.

Estimates of heart rate variability remained generally constant throughout the weekly classes, with only small areas of abnormally high or low variance. As mentioned, HRV is typically measured using medical evaluation technology that produces continuous heart rate tracking and allows for the analysis of the time lapses between heart beats. Studies that utilize technology that produces continuous heart rate tracking and can be employed in naturalistic settings may allow for more nuanced changes in HR and HRV to be revealed.

It may also be worthwhile to repeat the experiment with a focus on the interaction between the instructor and children during the lessons. As mentioned, visualization of the data showed a trend synchronization in heart rate among the children. Such heart rate synchronization has been documented in previous studies (Czepiel et al., 2021; Kragness et al., 2023; Pérez et al., 2021), and may have been due in part to the children’s concurrent attention to the musical stimuli (Kragness et al., 2023). In the future, gauging the extent to which the teacher’s heart rate synchronized along with children would provide useful context.

This preliminary study is limited by the small sample employed, and generalizations from this study would be inappropriate. Repeating the study with multiple classes of children would be instructive. Additionally, follow-up studies aimed at understanding children’s responses to music with varying levels of arousal and valence would be informative. Finally, in addition to studies investigating changes in heart rate between musical episodes that are familiar, unfamiliar, or transitional, we recommend that researchers study potential heart rate changes within musical episodes. That is, there may be cardiovascular changes that take place between the first iteration of a song or chant (which is unfamiliar), and the second iteration of a song or chant (which is more familiar). Analyzing these smaller windows of time and gradual shifts from the unfamiliar to the familiar, may shed light on cardiovascular responses while attending to musical stimuli, and the musical perception and cognition tendencies of young children.

## Data Availability

The raw data supporting the conclusions of this article will be made available by the authors, without undue reservation.

## Footnotes

1 Because these data were drawn from the first class, no indications of familiar material appear here.

2 Vertical gray lines indicate each of the songs/chants the children experienced during the class.
